# MicroRNA-153 targeting of KCNQ4 contributes to vascular dysfunction in hypertension

**DOI:** 10.1093/cvr/cvw177

**Published:** 2016-07-07

**Authors:** Georgina Carr, Vincenzo Barrese, Jennifer B. Stott, Oleksandr V. Povstyan, Thomas A. Jepps, Hericka B. Figueiredo, Dongling Zheng, Yalda Jamshidi, Iain A. Greenwood

**Affiliations:** af1 ^1^Vascular Research Centre, St George's University of London, Cranmer Terrace, London SW17 0RE, UK; af2 ^2^Ion Channels Group, Department of Biomedical Sciences, University of Copenhagen, Copenhagen, Denmark

**Keywords:** Arteries, Hypertension, KCNQ4, Kv7.4, miR153

## Abstract

**Aims:**

Kv7.4, a voltage-dependent potassium channel expressed throughout the vasculature, controls arterial contraction and is compromised in hypertension by an unknown mechanism. MicroRNAs (miRs) are post-transcriptional regulators of protein production and are altered in disease states such as hypertension. We investigated whether miRs regulate Kv7.4 expression.

**Methods and results:**

In renal and mesenteric arteries (MAs) of the spontaneously hypertensive rat (SHR), Kv7.4 protein decreased compared with the normotensive (NT) rat without a decrease in KCNQ4 mRNA, inferring that Kv7.4 abundance was determined by post-transcriptional regulation. *In silico* analysis of the 3′ UTR of KCNQ4 revealed seed sequences for miR26a, miR133a, miR200b, miR153, miR214, miR218, and let-7d with quantitative polymerase chain reaction showing miR153 increased in those arteries from SHRs that exhibited decreased Kv7.4 levels. Luciferase reporter assays indicated a direct targeting effect of miR153 on the 3′ UTR of KCNQ4. Introduction of high levels of miR153 to MAs increased vascular wall thickening and reduced Kv7.4 expression/Kv7 channel function compared with vessels receiving a non-targeting miR, providing a proof of concept of Kv7.4 regulation by miR153.

**Conclusion:**

This study is the first to define a role for aberrant miR153 contributing to the hypertensive state through targeting of KCNQ4 in an animal model of hypertension, raising the possibility of the use of miR153-related therapies in vascular disease.

## 1. Introduction

Primary hypertension is a major risk factor for cardiovascular disease which continues to be the leading cause of death globally and is predicted to dominate mortality trends in the future.^1^ The hypertensive state is precipitated by decreased luminal diameter brought about by increased arterial contractility, lack of response to endogenous vasorelaxants, and vessel wall remodelling.^2–5^ Among the possible modulators of arterial contractility, the Kv7 channels encoded by KCNQ genes regulate vascular smooth muscle contractility in rodent and human arterial beds, with Kv7.4 and Kv7.5 playing a key functional role in regulating vascular tone directly and contributing to endogenous vasodilatations.^3,4,6–15^ Recently, we discovered that Kv7.4 channel activity is down-regulated in aorta, mesenteric, renal, and coronary arteries from spontaneously hypertensive rats (SHRs) and mesenteric arteries (MAs) from angiotensin II-infused mice, which was associated with a reduction in total Kv7.4 protein.^3,4,6,7^ Interestingly, only in aorta from SHRs was a reduction in KCNQ4 mRNA detected, and cerebral arteries from SHRs exhibit no Kv7 dysfunction.^3,6,15^ These observations suggest that Kv7.4 abundance is determined by post-transcriptional mechanisms in an artery-specific manner.

MicroRNAs (miRs) are powerful post-transcriptional regulators of gene expression.^16,17^ These small (20–25 nt) noncoding RNAs bind to a target recognition site (‘seed sequence’) in the three prime untranslated region (3′UTR) of mRNA transcripts leading to mRNA degradation and/or inhibition/activation of protein translation depending on complementarity of the miR with the target mRNA.^18,19^ Several studies have determined the importance of miRs in vascular smooth muscle cell (VSMC) function in health and disease,^20–23^ but no study has examined the impact of miRs on vascular Kv7.4. Since miRs are heavily implicated in various vascular diseases and given the importance of Kv7 channels in the vasculature, this study investigates whether miRs have a regulatory role on Kv7.4 and identifies that miR153 targets KCNQ4, which may contribute to vascular dysfunction in hypertension.

## 2. Methods

Detailed experimental protocols are available in the Supplementary material online.

### 2.1 Animals

Male normotensive (NT) Wistar rats and SHRs (Charles River, U.K.; 147 ± 6 mmHg—mean BP in a cohort of 12-week SHRs), 12–16 weeks of age (175–225 g), were killed by cervical dislocation in accordance with the UK Animal (Scientific Procedures) Act 1986. All animals were culled by Schedule 1 methods, and therefore no approval from a local or university ethics review board was required. This investigation conforms to Directive 2010/63/EU of the European Parliament on the protection of animals used for scientific purposes.

### 2.2 *In silico* analysis for prediction of miR targets

Candidate miRs that potentially target the 3′UTR of KCNQ4 were identified using the established miR target prediction algorithm, TargetScan6.2 and mirDIP, a miR data integration portal.

### 2.3 Transfection of miR mimics into mesenteric or middle cerebral arteries

Synthetic RNA molecules, miR153, miR133a, and non-targeting control (NTC) miR (Active Motif, La Hulpe, Belgium), were designed to mimic endogenous mature miR153, miR133a, or act as a NTC, respectively. NTC miR has no known homology to human (or rat) gene sequences and acts as a negative control. Whilst miR153 acts as a functional equivalent to endogenous human miR153, the sequence is 100% conserved in rat. Twelve-nanomolar miR153 (+ miR133a for cotransfection studies) or NTC miR were transfected into mesenteric or middle cerebral arteries (MCAs) from NT rats using *Trans*IT-X2 and OptiMEM® I Reduced-Serum Medium according to the manufacturer's protocol (Mirus Bio LLC, Madison, USA). Twenty-four hours post transfection, arteries were used in functional/staining studies or harvested for RNA or protein for quantitative polymerase chain reaction (qPCR) or western blot, respectively.

### 2.4 Synthesis of KCNQ4 3′UTR luciferase reporter, miR153 binding site mutants and transfection into HEK293 cells

A fragment of the KCNQ4 mRNA 3′UTR (NM_0047000.3) containing 4 predicted binding sites for miR153 was synthesized and subcloned into pLightSwitch_3′UTR luciferase expression reporter vector, downstream of the *Renilla* luciferase reporter gene (GoClone), by SwitchGear Genomics (Active Motif). Site-specific mutagenesis of two predicted miR153 binding sites was engineered in pLightSwitch-KCNQ4_3′UTR vector using the QuikChange II site-directed mutagenesis kit (Agilent Technologies, CA, USA) according to the manufacturer's instructions. Deletion of seed sequences corresponding to Site 1 (Δ Site 1, 8 nucleotides) and Site 4 (Δ Site 4, 7 nucleotides) was obtained using specific pairs of primers designed using the online QuikChange Primer Design Program (www.agilent.com/genomics/qcpd). Effective deletion of Site 1 and Site 4 regions was verified by direct sequencing. The pLightSwitch_3′UTR luciferase expression reporter vector containing no insert (EMPTY_3UTR, Active Motif) was used as positive control for transfection to allow assay normalization. The following groups were set up for transfection of HEK293 cells using *Trans*IT-X2: (i) WT KCNQ4 3′UTR + miR153, (ii) WT KCNQ4 3′UTR + NTC miR, (iii) Δ Site 1 KCNQ4 3′UTR + miR153, (iv) Δ Site 1 KCNQ4 3′UTR + NTC miR, (v) Δ Site 4 KCNQ4 3′UTR + miR153, (vi) Δ Site 4 KCNQ4 3′UTR + NTC miR, (vii) WT KCNQ4 3′UTR, (viii) EMPTY_3′UTR, and (ix) *Trans*IT-X2. Twenty-four hours post transfection, luciferase activity was measured using the LightSwitch Assay Reagent kit (LS010, Active Motif) designed for use with all GoClone reporter plasmids.

### 2.5 RNA extraction, reverse transcription, and quantitative polymerase chain reaction

The miRNeasy Mini Kit (Qiagen, Manchester, UK) was used for effective purification of miR and total RNA from dissected rat arteries. Samples were reverse transcribed using the miScript II RT Kit (Qiagen) with HiFlex buffer to allow miR as well as mRNA to be quantified from the same sample. Measurements of miR or mRNA levels were performed by qPCR using the miScript SYBR Green PCR Kit (Qiagen) with the CFX96 real-time PCR Detection System (Bio-Rad, Hertfordshire, UK).

### 2.6 Western blot

Artery protein lysates were probed after transfer to polyvinylidene fluoride (PVDF) membrane (Millipore, Hertfordshire, UK) with a primary antibody diluted in blocking buffer either overnight at 4°C or for 1 h at room temperature. Primary antibodies used were rabbit anti-Kv7.4 (1:200; sc-50417; Santa Cruz, TX, USA) and mouse β-Actin (1:5000; A1978; Sigma Aldrich, Dorset, UK).

### 2.7 Functional studies

Morphological properties of MAs from NT rats and SHRs as well as NT MAs treated with NTC miR or miR153 were studied using Pressure Servo System PS/200 (Living Systems Instrumentations, Burlington, VT, USA). The structural properties of the segments and responses to the Kv7 activator, S1 (NeuroSearch A/S, Ballerup, Denmark) were recorded and acquired with DMK 41AU02 Monochrome Industrial Camera (Imaging Source, Bremen, Germany) hosted by a PC running MyoVIEW II software (Danish Myo Technology, Aarhus, Denmark). Data were analysed using MyoVIEW II and MicroCal Origin 6.0 (MicroCal Software, Northampton, MA, USA). Isometric tension was recorded on NT MAs or MCAs transfected with either mir153 or NTC miR in a wire myograph (Danish Myo Technology). Mesenteric vessels were pre-constricted with 1 µM U46619 (Sigma Aldrich) and a concentration effect curve to the Kv7.2–7.5 activators ML213 (HelloBio, Bristol, UK), ICA-069673 (HelloBio), the β-adrenoceptor agonist isoprenaline (Sigma Aldrich), or the Kv7.1-specific activator RL-3 (Tocris, Bristol, UK) as well as a relaxation response to 1 µM nicardipine (Sigma Aldrich) were obtained. Relaxant responses to S1 or ML213 were also assessed in MCAs transfected with miR153 or NTC and pre-constricted with 100 nM U46619. Data were recorded and analysed using LabChart® 7 (ADInstruments, Dunedin, New Zealand).

### 2.8 Histology

MA branches transfected with NTC miR or miR153 were embedded in OCT, frozen rapidly, and cryosectioned at ∼7 µm. Slices were then fixed with ice-cold acetone and stained using an Elastica van Gieson staining kit or successively stained with haematoxylin and eosin (H&E; Merck, NJ, USA). After dehydration, slices were covered with DPX mounting solution and stored for further use. An Axioplan 2 Upright microscope with attached Axio Cam Digital CCD camera (Carl Zeiss Ltd, Cambridge, UK) was used for imaging.

### 2.9 Statistical analysis

The data were expressed as mean ± SEM (standard error) with *N* representing the number of animals and *n* the number of experiments. Comparisons of data were accomplished by one-way or two-way ANOVA followed by Bonferroni's multiple comparisons test; Student's *t*-test, or paired *t*-test as appropriate. The differences between means were considered significantly different when *P* < 0.05.

## 3. Results

### 3.1 Kv7.4 protein is down-regulated in hypertensive arteries

**Figure 1:**
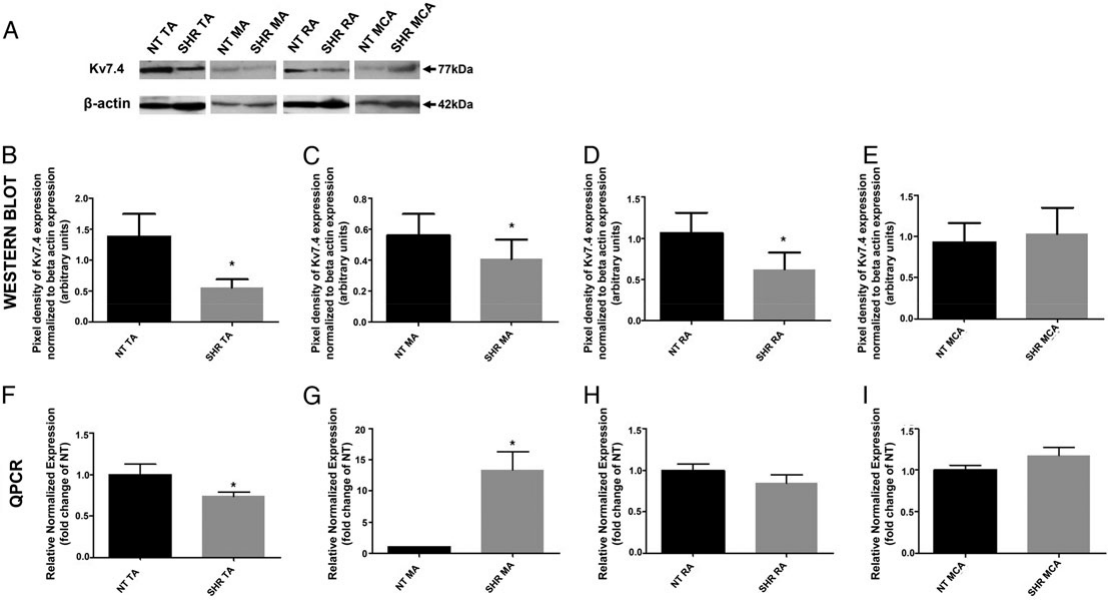
Kv7.4 protein expression is decreased in SHR mesenteric, renal, and thoracic aortic arteries compared with NT rats with a decrease in KCNQ4 expression in SHR thoracic aortic arteries compared with NT rats. (*A*) Representative western blot for Kv7.4 expression in total protein lysates from NT rats and SHRs: TA, MA, RA, and MCA. β-Actin bands indicate protein loading controls. (*B–E*) Mean (±SEM) pixel densities of Kv7.4 bands normalized to their respective β-actin bands for NT and SHR (*B*) TA, (*C*) MA, (*D*) RA, and (*E*) MCA from at least 3 separate protein isolations (*N* = 3–8). (*F–I*) QPCR analysis with 2^−ΔΔCq^ of relative abundance of KCNQ4 of the SHR compared with the NT rat in (*F*) TA, (*G*) MA, (*H*) RA, and (*I*) MCA from at least 3 separate RNA isolations (mean ± SEM, *N* = 3–4). *P < 0.05 according to paired Student's *t*-test.

Previous findings on Kv7.4/KCNQ4 arterial expression in hypertensive animals have been obtained in different arteries in various separate studies.^3,4,6,7,15^ Initial experiments in the current study therefore sought to corroborate these previous results but within one study to allow novel comparisons to be made. Interrogation of protein lysates from HEK293 cells stably expressing Kv7.4, with a Kv7.4 antibody produced reactive bands corresponding to the theoretical molecular weight of Kv7.4 that were not present in untransfected HEK293 cells (*n* = 3, Supplementary material online, *Figure S1*). Similar reactive bands were identified in protein lysates from thoracic aortic arteries (TAs), MAs, renal arteries (RAs) and MCAs from NT rats and SHRs (*Figure 1A*). Kv7.4 expression was decreased in TAs (59.45 ± 15.69%, *N* = 8, *P* = 0.02), MAs (27.33 ± 8.32%, *N* = 8, *P* = 0.04), and RAs (36.14 ± 11.96%, *N* = 6, *P* = 0.03) from SHRs compared with NT rats (*Figure 1B–D*). In contrast, there was no difference in Kv7.4 protein expression in MCAs from SHR and NT animals (*N* = 3, *P* = 0.21, *Figure 1E*).

QPCR was undertaken to assess whether the decrease of Kv7.4 protein in SHRs correlated to a reduction of KCNQ4 mRNA. In TAs from SHRs, there was a 1.35-fold decrease in KCNQ4 mRNA expression (*N* = 4, *P* = 0.03, *Figure 1F*), whereas for MAs, KCNQ4 mRNA increased 13.35-fold (*N* = 3, *P* = 0.02, *Figure 1G*) in SHRs compared with NT rats. There was no difference in mRNA levels between SHRs and NT rats in RAs (*N* = 4, *P* = 0.29, *Figure 1H*) or MCAs (*N* = 3, *P* = 0.17, *Figure 1I*). Overall, these data show that Kv7.4 is compromised in different arteries from SHRs that is not consistently associated with a change in transcript level.

### 3.2 miR153 increases in SHR mesenteric, renal, and thoracic aortic arteries compared with NT rats

TargetScan6.2 and mirDIP were used to identify candidate miRs targeted at the 3′UTR of KCNQ4 (Supplementary material online, *Figure S2*). This analysis revealed putative seed sequences in KCNQ4 for miR26a, miR214, miR133a, miR200b, miR153, miR218, and let-7d. Among them, miR153 showed the highest probability of consensus targeting. Analogous miR153 seed sequences were not detected in the 3′ UTR of KCNQ5 or KCNQ1, the other KCNQs highly expressed in the vasculature.^8^

**Figure 2:**
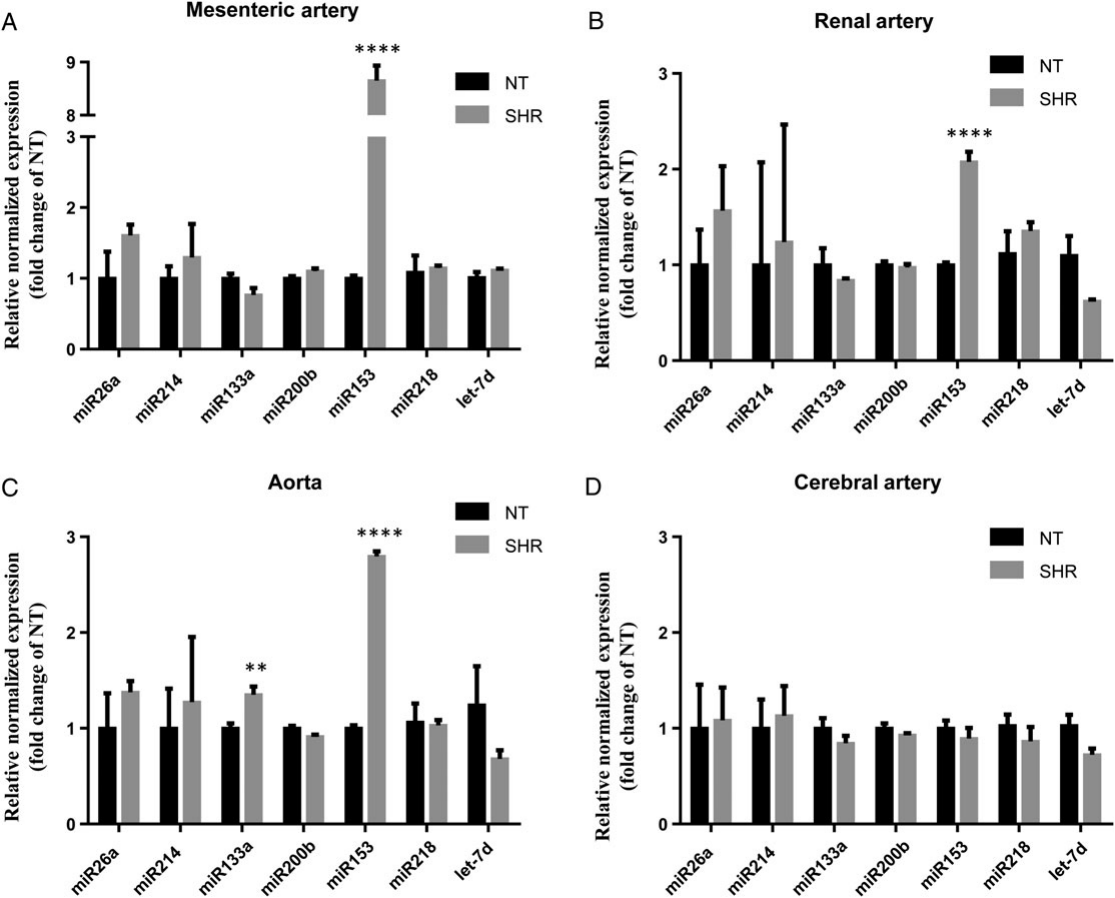
miR153 increases in SHR mesenteric, renal, and thoracic aortic arteries compared with NT rats. QPCR analysis with 2^−ΔΔCq^ of relative abundance of miR26a, miR214, miR133a, miR200b, miR153, miR218, and let-7d of the SHR compared with the NT rat in (*A*) MA, (*B*) RA, (*C*) TA, and (*D*) MCA from at least 5 separate RNA isolations (mean ± SEM, *N* = 5–6). ****P < 0.0001 and **P < 0.01 according to Student's *t*-test.

The working hypothesis is that an increase in miR expression in SHR arteries results in Kv7.4 reduction. *Figure 2* shows that whilst the expression of miR26a, miR200b, miR214, miR218, and let-7d was not altered in arteries from SHRs compared with NT, a significant increase of miR153 was recorded in SHR MA (8.65, *N* = 6, *P* < 0.0001), RA (2.08, *N* = 6, *P* < 0.0001), and TA (2.80, N = 6, *P* < 0.0001). A 1.35-fold increase was also observed for miR133a in SHR aorta (*N* = 6, *P* = 0.006). Interestingly, miR153 was higher in the plasma from SHRs compared with NT plasma (*N* = 6, *P* = 0.14, Supplementary material online, *Figure S3*). In contrast, there was no change in the expression of any miRs in MCAs from SHRs compared with NT rats (*Figure 2D*, *N* = 5, all *P*-values were not significant). Consequently, miR153 expression paralleled hypertension-induced changes in Kv7.4 levels across the arteries under study.

### 3.3 miR153 is confirmed to interact functionally with KCNQ4 3′UTR

**Figure 3:**
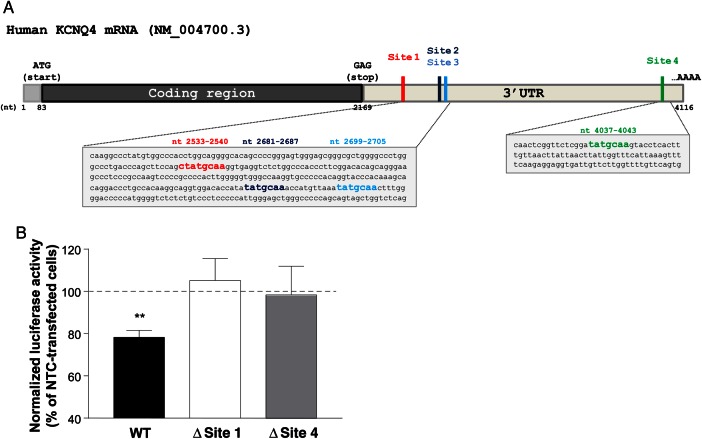
miR153 targets KCNQ4 3′UTR. (*A*) Schematic representation of human KCNQ4 mRNA 5′UTR, coding sequence, and 3′UTR with miR153 binding sites highlighted. Red, blue, light blue, and green squares in the 3′UTR indicate the predicted binding sites for miR153 (Sites 1, 2, 3, and 4, respectively). Seed sequences corresponding to Site 1 (red), Site 2 (blue), Site 3 (light blue), and Site 4 (green) are highlighted in the grey boxes showing the detailed nucleotide sequence of KCNQ4 3′UTR. Positions of the seed sequences are indicated above with the same colour scheme. (*B*) Mean luciferase activity in HEK293 cells of KCNQ4 3′UTR WT, with deleted Site 1 (Δ Site 1), and with deleted Site 4 (Δ Site 4), co-transfected with miR153. Data are normalized to HEK293 cells transfected with KCNQ4 3′UTR only and expressed as % of HEK293 cells transfected with the same plasmids in the presence of NTC (mean ± SEM, *n* = 3–4). **P < 0.01 according to paired Student's *t*-test (miR153 vs. NTC for each plasmid).

A luciferase reporter was engineered to contain a fragment of the KCNQ4 mRNA 3′UTR encompassing 4 predicted binding sites for miR153 (wild-type, WT); or deletion of seed sequences corresponding to Site 1 (Δ Site 1) and Site 4 (Δ Site 4, *Figure 3A*). HEK293 cells cotransfected with WT KCNQ4 3′UTR and miR153 produced a decrease in luciferase activity compared with those cotransfected with KCNQ4 3′UTR and the non-targeting miR control (NTC miR, *n* = 4, *P* < 0.01, *Figure 3B*), which was eliminated when the 2 predicted binding sites were deleted (Δ Site 1, *n* = 3, *P* = 0.72 and Δ Site 4, *n* = 4, *P* = 0.76); indicating that miR153 interacts functionally with the 3′UTR of KCNQ4.

### 3.4 Addition of a synthetic miR153 to NT mesenteric arteries mimics an SHR-like phenotype

**Figure 4:**
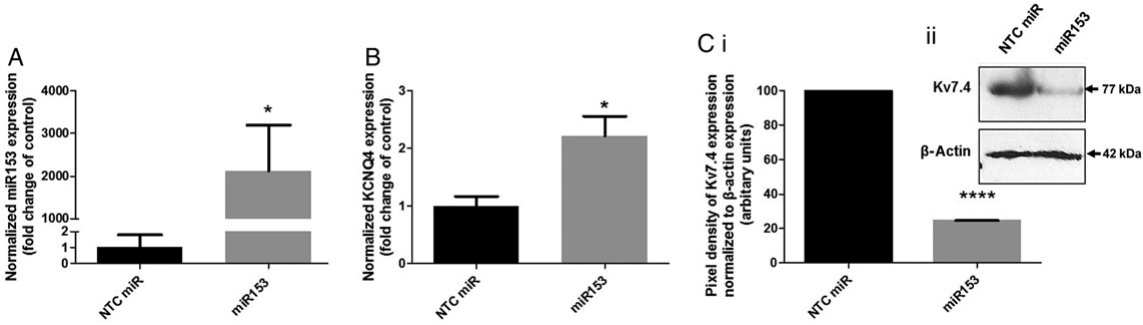
Transfection of NT MAs with miR153 leads to up-regulation of KCNQ4 expression and down-regulation of Kv7.4 expression. QPCR analysis with 2^−ΔΔCq^ of relative abundance of (*A*) miR153 or (*B*) KCNQ4 in NT MAs transfected with non-targeting miR control (NTC) or miR153; data are normalized to NTC-transfected NT MAs (mean ± SEM, *N* = 11 and *N* = 4, respectively). *P < 0.05 according to paired Student's *t*-test. (*Ci*) Mean pixel densities of Kv7.4 bands normalized to their respective β-actin bands for NT MAs transfected with NTC or miR153 from 3 separate protein isolations (*N* = 3). ****P < 0.0001 according to ratio paired *t*-test. (*Cii*) Representative western blot for Kv7.4 expression in total protein lysates from NT MAs transfected with NTC or miR153. β-Actin bands indicate control for protein loading.

In all of the arteries studied, SHR MAs showed the greatest fold increase in miR153 compared with NT rats; hence, we focused on MAs from NT rats for our transfections studies with a miR153 mimic. Addition of miR153 to MAs from NT rats resulted in a large increase in miR153 levels compared with arteries from the same animal transfected with NTC miR (*N* = 11, *P* = 0.04, *Figure 4A*). In arteries transfected with miR153, KCNQ4 mRNA expression increased approximately two-fold (*N* = 4, *P* = 0.02, *Figure 4B*), whilst Kv7.4 abundance decreased by 75.43 ± 0.15% (*N* = 3, *P* < 0.0001, *Figure 4C*) compared with NTC miR-transfected vessels, which mirrored the changes recorded for SHRs shown in *Figure 1*. Cotransfection of NT MAs with miR153 and miR133a showed a marked decrease of KCNQ4 (*N* = 4, *P* < 0.05, Supplementary material online, *Figure S4**A*) but not KCNQ5 (*N* = 4, *P* = 0.34, Supplementary material online, *Figure S4**B*), compared with NTC miR-transfected vessels.

**Figure 5:**
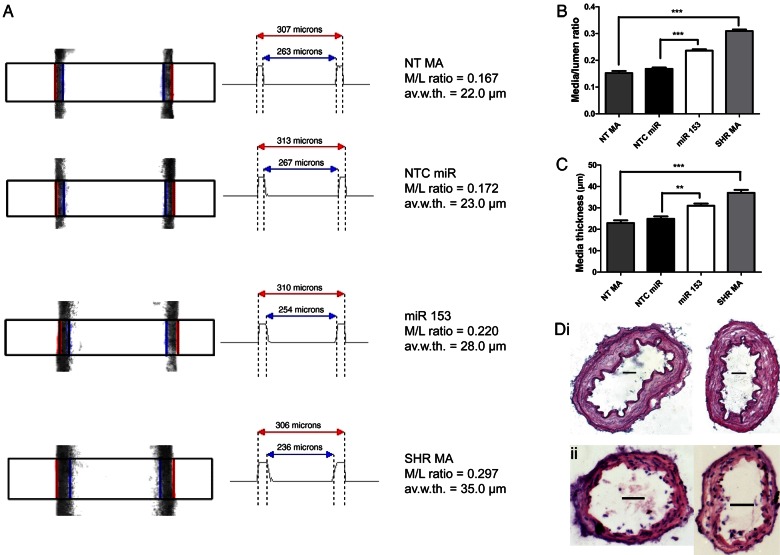
Addition of miR153 to MAs of NT rats leads to vascular wall thickening. (*A*) Examples of raw CCD camera images (left) and corresponding automatic digitizing of outer and inner diameters (right) of native rat MA segments pressurized to 60 mmHg for NT MA, non-targeting miR control (NTC miR)-transfected artery, miR153-transfected artery (miR153), and SHR MA. (*B*) Mean (±SEM) media/lumen ratios and (*C*) mean (±SEM) media thickness of NTC miR compared with miR153-transfected NT MAs; and MAs of NT rats compared with SHRs (*n* = 10–12; *N* = 7–10). ***P < 0.001 or **P < 0.01 according to one-way ANOVA followed by Bonferroni's multiple comparisons test. (*D*) Representative images of (i) Elastica van Gieson staining or (ii) H&E staining (dark blue indicates nuclei, *n* = 12–13) of NTC miR (left image) and miR153 (right image) transfected MA segments; scale bars: 35 µm.

Perfusion myograph experiments showed that transfection of MAs from NT rats with miR153 produced morphological changes to the artery that approximated a SHR-like phenotype (*Figure 5A–C* and Supplementary material online, *Tables S1* and *S2*, *n* = 10–12; *N* = 7–10). In all experiments, arteries with similar outer wall dimensions were used, and transfection with NTC miR had no effect on wall morphology (*Figure 5*, Supplementary material online, *Tables S1* and *S2*). However, transfection with miR153 for 24 h produced a significant increase in the media/lumen ratio that approached the values obtained in arteries from SHRs (*Figure 5A* and *B*, Supplementary material online, *Tables S1* and *S2*). Average wall thickness was also enhanced in miR153-transfected (31.0 ± 1.0 µm) and SHR (37.0 ± 1.4 µm) MAs compared with NTC miR-transfected vessels (24.9 ± 1.1 µm, *P* < 0.01) or NT vessels (22.9 ± 1.3 µm, *P* < 0.001), respectively (*Figure 5A* and *C*, Supplementary material online, *Tables S1* and *S2*) with *Figure 5Di* indicating an increase in the *tunica media* of miR153-transfected vessels compared with the control but no increase in cell number (*Figure 5Dii*; 49 ± 2 vs. 47 ± 3 nuclei per slice for NTC miR- or miR153-transfected NT MAs, respectively, *n* = 12–13, *P* = 0.621). Consequently, a short-term increase of miR153 in NT MAs produced morphological changes analogous to arteries from SHRs.

**Figure 6:**
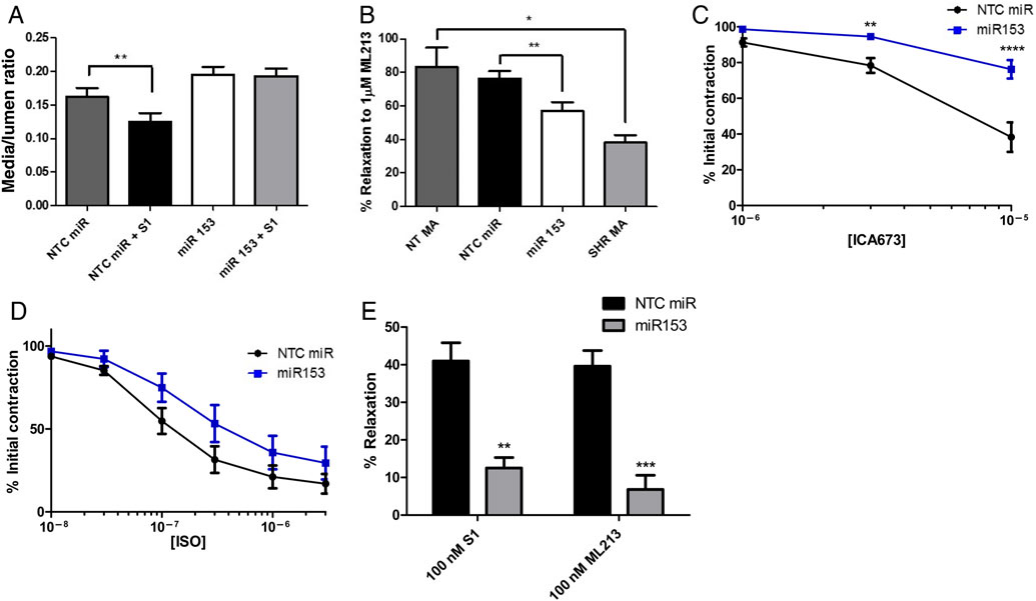
Addition of miR153 to MAs or MCAs of NT rats leads to a reduced response to Kv7 activators. (*A*) Mean (±SEM) media/lumen ratios of non-targeting miR control (NTC miR)-transfected NT MAs compared with S1-treated NTC miR-transfected vessels; and miR153-transfected MAs compared with S1-treated miR153-transfected NT MAs (*N* = 4). (*B*) Mean (±SEM) percentage relaxation to 1 µM ML213 from isometric tension recordings in NTC miR compared with miR153-transfected NT MAs; and in MAs of NT (NT MA) rats compared with SHR MA (*N* = 3–8). *P < 0.05 and **P < 0.01 according to one-way ANOVA followed by Bonferroni's multiple comparisons test. Isometric tension recordings of relaxation to increasing concentrations of (*C*) the Kv7 activator ICA-069673, (*D*) the β-adrenoceptor agonist isoprenaline in NT MAs transfected with miR153 or NTC miR. Each point represents the mean of 4–8 animals ± SEM. ****P < 0.0001 and **P < 0.01 according to two-way ANOVA followed by Bonferroni's multiple comparisons test. (*E*) Mean (±SEM) percentage relaxation to 100 nM S1 or 100 nM ML213 from isometric tension recordings in NTC miR compared with miR153-transfected NT MCAs (*N* = 4–5). ***P < 0.001 and **P < 0.01 according to two-way ANOVA followed by Bonferroni's multiple comparisons test.

In further perfusion myograph studies application of the Kv7 activator, S1 caused a decrease in the media/lumen ratio in NTC miR-transfected NT vessels (0.162 ± 0.013–0.125 ± 0.013, *N* = 4, *P* < 0.01) but had no effect on miR153-transfected MAs (0.195 ± 0.012 ± S1 vessels, *N* = 4, *P* = 0.391, *Figure 6A*). Isometric tension recordings of these miR153-transfected vessels also showed decreased relaxation to two structurally different Kv7 activators, ML213^24^ (*N* = 8, *P* < 0.01, *Figure 6B*), which was similarly lacking in arteries from SHRs (*N* = 3, *P* < 0.05 *Figure 6B*); and ICA673 (*N* = 5, *P* < 0.0001 or *P* < 0.01, *Figure 6C*). A similar trend in the relaxation response to the β-adrenoceptor agonist isoprenaline was also observed in miR153-transfected MAs (*N* = 6, *P* > 0.05, *Figure 6D*). Neither the relaxant effect of 1 µM nicardipine (86.98 ± 0.68 vs. 90.53 ± 2.68 for NTC miR- or miR153-transfected NT MAs, respectively, *N* = 4–5, *P* = 0.3) and the Kv7.1-specific activator, RL-3^25^ (*N* = 4–8, Supplementary material online, *Figure S5**A*) nor vessel constriction by the thromboxane A2 analogue U46619 (*N* = 4–8, Supplementary material online, *Figure S5**B*) was affected by miR153 over-expression. Consequently, increasing miR153 levels exogenously to NT MA mimics functional characteristics of MAs from SHRs. Interestingly, in cerebral arteries where responses to Kv7.2–5 activators are unaffected in SHRs^15^ and no rise in miR153 was detected, transfection with exogenous miR153 produced a marked impairment in the relaxant response to S1 and ML213 (*N* = 4–5, *P* < 0.01 and *P* < 0.001, respectively, *Figure 6E*).

## 4. Discussion

This study is the first to provide evidence for a role for miRs in the regulation of Kv7.4 in the vasculature and raises the possibility that this post-transcriptional regulation is a contributing factor of vascular dysfunction. As there is considerable evidence that Kv7 channels regulate vascular smooth muscle contractility and contribute to the vasodilator response of many endogenous agents,^3,4,6–15,24,26–28^ the present study implicates miR153 as a determinant of vascular tone and potentially a novel therapeutic target for the treatment of hypertension.

In accordance with previous findings,^3,4,7^ we observed a decrease in Kv7.4 protein expression in mesenteric and renal arteries from SHRs that was not associated with a decrease of KCNQ4 mRNA. This suggests that a post-transcriptional regulator, such as miRs, dictates Kv7.4 abundance. Indeed, the expression profile of miRs predicted to target KCNQ4 revealed an up-regulation of miR153 in arteries from SHRs compared with NT rats. Moreover, in cerebral arteries, where there was no difference in Kv7.4 protein or mRNA expression between NT and SHR and no change in Kv7 activity functionally,^15^ there was no increase of miR153 or any of the miRs analysed. Hence, the miR153 expression profile correlates closely with the Kv7.4/KCNQ4 expression profiles for the four arteries in hypertension. The reason for this variability between arteries is difficult to define in detail since the nature of cell-specific regulation by miRs continues to be investigated, but it provides a mechanism to tailor cellular responses to individual cell types. We propose that miR153 represses translation of KCNQ4 mRNA rather than by transcript degradation as KCNQ4 levels did not decrease in renal or MAs. In fact, KCNQ4 expression increased markedly in MAs from SHRs or after miR153 transfection, which is probably due to the suppression of a negative feedback loop altering transcription factors.^29^ In aorta from SHRs, miR133a as well as miR153 increased, but in this artery, the decreased Kv7.4 protein expression was associated with a decrease in mRNA. Interestingly, we reduced KCNQ4 transcript levels in NT MAs by co-transfecting miR153 with miR133a providing support for our hypothesis that in the aorta a rise in miR133a affects KCNQ4 transcription, whereas the rise in miR153 seen in the aorta, renal, and MAs leads to impaired translation of KCNQ4 mRNA. Mir133a has been implicated in modulation of the VSMC phenotype in general and specifically in the regulation of VSMC proliferation in mouse aorta.^23,30^ However, as miR133a was unchanged in other hypertensive vessels that displayed reduced Kv7.4 expression and miR153 was consistently up-regulated in these vessels, we focused on defining the impact of miR153 on Kv7.4 in hypertension.

Having identified a rise in miR153 expression correlated with a reduction of Kv7.4, we established that miR153 has a direct targeting effect on the 3′UTR of KCNQ4 using a luciferase reporter assay. Furthermore, we showed that addition of synthetic miR153 to MAs from NT rats caused an up-regulation of KCNQ4 mRNA, decreased Kv7.4 protein expression, and attenuated relaxations to structurally different Kv7 activators similar to that seen in arteries from SHRs (current study and^3,4,7,15^). A shift in the relaxation response to the β-adrenoceptor agonist isoprenaline was also observed in miR153-transfected MAs although not significantly, which may reflect the complex intracellular signalling involved. Interestingly, transfection of miR153 impaired the relaxation induced by Kv7 activators in the cerebral artery, which showed no change in miR153 levels between NT and hypertensive rats, further suggesting that a rise in miR153 down-regulates Kv7.4 protein function. It is worth stressing that the rise in miR153 produced by transfection with a synthetic mimetic far exceeds levels seen in arteries from SHRs. However, we have no knowledge about the regulation of miR153 in arteries in response to hypertensive cues, and the data are a good proof of concept. The striking similarity in Kv7 characteristics between arteries from SHRs and those from NT rats transfected with miR153 provides strong support for miR153 regulation of Kv7.4. Furthermore, it is possible that in SHRs, miRs exert their effect in a time-dependent manner. Thus, to obtain a similar effect in our *in vitro* system, after only 24 h, we need a very high concentration of miR153.

Remarkably, our pressure myography data showed that media/lumen ratio and average wall thickness were significantly increased in miR153-transfected vessels compared with NTC miR-transfected vessels, showing a tendency of shifting the morphological characteristics of native NT MAs towards an SHR phenotype within 24 h after transfection. The increase in average wall thickness (from 24.9 ± 1.1 to 31.0 ± 1.0 µm) produced by miR153 transfection is equivalent to the possible appearance of just one extra layer of smooth muscle cells. Vascular remodelling^31,32^ involves several cell types including a phenotypic switch of VSMCs between a differentiated, contractile state with a low proliferation rate to a dedifferentiated, synthetic state in which VSMCs are more proliferative.^33,34^ Studies with vascular smooth muscle-specific knockout of Dicer show miRs are crucial for VSMC development, differentiation, and contractile function.^20–22,35^ Importantly, widespread loss of miR expression in these knockout animals caused decreased blood pressure attributed to a reduced contractile tone and media thickness in aorta.^36^ miR153 has also been linked to proliferation in venous samples after mechanical injury.^37^ However, H&E staining did not reveal an increase in cell number in the tunica media, suggesting that the wall thickening stems from deposition of collagen, fibronectin, and other components of the extracellular matrix similar to the remodelled arterial structures in SHRs^31,32^ or there is an increase in cell size. The mechanisms linking Kv7.4 dysfunction and changes in arterial morphology and responsiveness will be the focus of future studies.

It should be emphasized that miR153-induced KCNQ4 down-regulation is only a contributory component in the multifactorial disease of hypertension. miR153 is known to affect targets other than KCNQ4,^37–40^ although TargetScan6.2 predicted miR153 to only target the 3′UTR of KCNQ4 and not KCNQ5 or KCNQ1, the other KCNQs highly expressed in the vasculature. Moreover, KCNQ1 and KCNQ5 are down-regulated by other miRs, namely, miR1/133 and miR190, respectively.^41,42^ Nevertheless, the parallel change in miR153 and Kv7.4 is clear raising the possibility that correction of the miR153 up-regulation that leads to the aberrant Kv7 channel function/expression, vasoconstriction, and vascular wall thickening, through the use of miR sponges,^43^ chemical inhibitors,^44^ and antagomirs^45^ as a potential treatment for hypertension. Furthermore, a rise of miR153 was detected in the blood of SHRs, suggesting that this may present a novel diagnostic marker for hypertensive scenarios. However, investigation of antagomiRs on *ex vivo* arteries from SHRs should be approached with caution since we have preliminary data indicating that a ‘loss’ of the SHR phenotype occurs in arteries incubated in Dulbecco's modified Eagle's medium (DMEM) for 24–72 h (Supplementary material online, *Figure S6**A*–*C*). Interestingly, we observed a decreased trend of miR153 in MAs from SHR upon incubation in DMEM for 48 h, possibly explaining the loss of Kv7.4 functional impairment observed in the incubated vessels (Supplementary material online, *Figure S6**D*).

Increased vascular tone, a relative resistance to vasorelaxants and vascular remodelling underlie vascular disease. Our data define a role for aberrant miR153 underlying this hypertensive state through targeting of KCNQ4 in an animal model of hypertension. Research into vascular Kv7 channels is in its infancy, and therefore it is not possible at the moment to determine how much regulation of Kv7.4 channels by miRs contributes to post-transcriptional control globally. Further research is required to determine the full spectrum of miR153 target genes, possible side effects, and a therapeutic strategy/delivery for the correction of miR153 up-regulation, but the potential use of miR153-related therapies in clinical vascular disease is certainly worth exploring further.

## Supplementary material

Supplementary material is available at *Cardiovascular Research* online.

## Funding

This work was supported by the Medical Research Council (MR/K019074/1 to G.C. and O.V.P.), the British Heart Foundation (PG/12/63/29824 to J.B.S.), and the People Programme (Marie Curie Actions) of the European Union's Seventh Framework Programme, FP7/2007-2013 (Research Executive Agency grant agreement no. 608765 to T.A.J.). Funding to pay the Open Access publication charges for this article was provided by the Medical Research Council (80%) and the British Heart Foundation (20%).
